# Supplementary material for: Treeline displacement may affect lake dissolved organic matter processing at high latitudes and altitudes
Source: Nat Commun. 2024 Mar 26;15:2640. doi: 10.1038/s41467-024-46789-5 (PMC10965997; doi:10.1038/s41467-024-46789-5)
Supplement: Supplementary file 1 — Supplementary Information [file 41467_2024_46789_MOESM1_ESM.pdf]

SUPPLEMENTARY INFORMATION

Treeline displacement may affect lake dissolved organic matter processing at high latitudes and altitudes

Núria Catalán[1,2*], Carina Rofner[3], Charles Verpoorter[4], María Teresa Pérez[3], Thorsten Dittmar[5,6], Lars Tranvik[1], Ruben Sommaruga[3] and Hannes Peter[3,7*]

[1]Limnology, Department of Ecology and Genetics, University of Uppsala, Uppsala, Sweden
[2]Present address: Institute of Environmental Assessment and Water Research (IDAEA), CSIC, Barcelona, Spain
[3]Lake and Glacier Ecology Research Group, Department of Ecology, Universität Innsbruck, Innsbruck, Austria
[4]Université Littoral Cote d'Opale, CNRS, Université Lille, UMR 8187, Laboratoire d'Océanologie et de Géosciences, Wimereux, F629300 Wimereux, France
[5]Institute for Chemistry and Biology of the Marine Environment (ICBM), University of Oldenburg, Oldenburg, Germany
[6] Helmholtz Institute for Functional Marine Biodiversity (HIFMB) at the University of Oldenburg, Oldenburg, Germany
[7] River Ecosystems Laboratory, École Polytechnique Fédérale de Lausanne (EPFL), Lausanne, Switzerland

**corresponding authors: NC: nuria.catalan@idaea.csic.es; HP: hannes.peter@epfl.ch*

INDEX

S. I. 1.- Optical spectroscopy and PARAFAC model

S. I. 2.- Reactivity continuum models on bulk DOC

S. I. 3.- FT-ICR MS data treatment

S. I. 4.- Microbial respiration

Supplementary Information 1. Optical spectroscopy and PARAFAC model

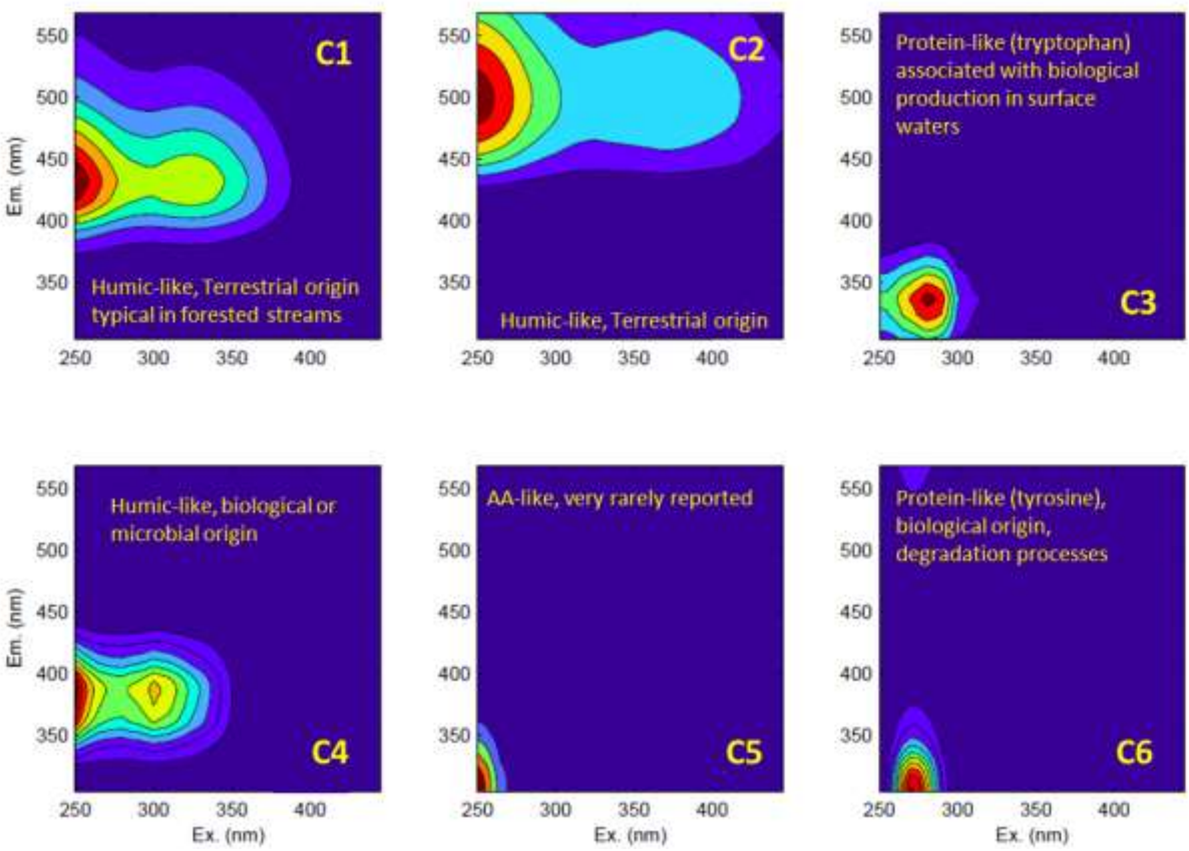

Supplementary Figure S1. Fingerprints of the 6 components identified in the Parallel FACtor analaysis (PARAFAC) model. The model is available at the OpenFluor database under accession number: ID 18555.

Detail on methods: PARAFAC modelling and interpretation

Scatter peaks and outliers were removed and each sample normalized to its total fluorescence prior to fitting a PARAFAC model. The appropriate number of components was determined by visual inspection of the residuals and of the components' behavior as organic fluorophores[1,2]. The model was then validated by split-half analysis and random initialization with 10 iterations. We interpreted the components based on their fluorescence maxima, and compared them (TCC = 96%) with the matched emission and excitation spectra in the OpenFluor database of components previously identified in natural aquatic systems (https://openfluor.lablicate.com/ in December 2023), in order to search for quantitative matches with previously published and validated PARAFAC models[3]. Six components were identified. Humic-like components C1, C2 and C4 matched 37, 165 and 9 published PARAFAC models respectively. C1 signal has been linked to terrestrial sources in forested catchments and found across seawater, freshwater and artificial systems[4,5,6]. C2 is related with aromatic and colored materials in, e.g. boreal lakes[7]. C4 is related to peak M and processed humic materials, and it has also been related with protein-like materials and microbial activity[8,9]. Components C3, C5 and C6 corresponded to tryptophan-like and tyrosine-like fluorophores. C3 matched 52 models, expected to be from biological in-situ sources, it appears associated with aliphatic and phenolic constituents (S.I. Figure S2). C5 is rarely

reported (matched 3 models) and sometimes has been related with leaf-litter leachates[10], here it is clearly related with unsaturated and highly unsaturated constituents (S.I. Figure S2). Similarly, C6, matching 14 models corresponds with a protein-like, linked to a biological origin or degradation processes.

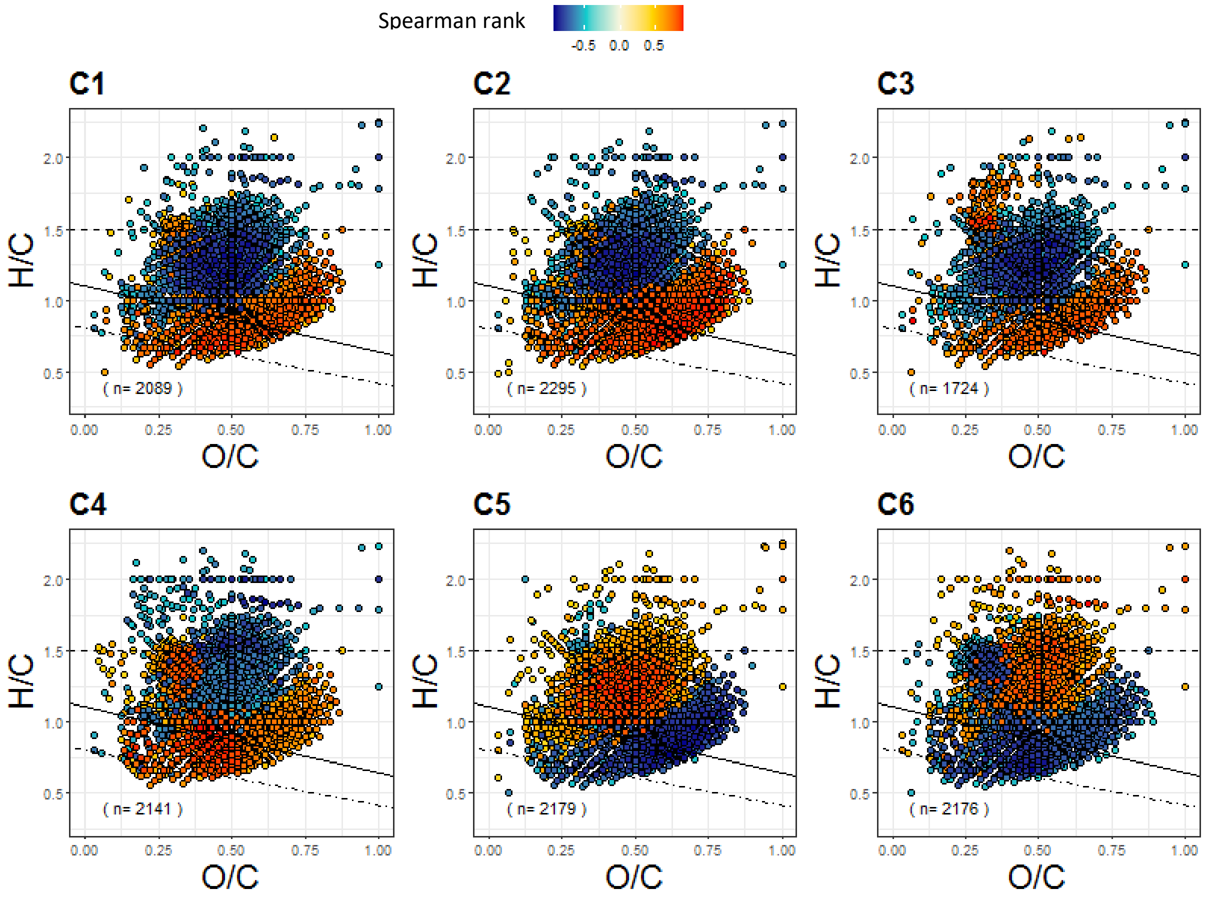

Supplementary Figure S2. Van Krevelen diagrams showing significant ($p<0.05$) Spearman rank correlations between individual compound intensity and PARAllel FACtor analysis (PARAFAC) components. Significance of Spearman correlations have been corrected for multiple comparisons. Lines separate compound groups as in Figure 1 (main text).

Supplementary Information 2. Reactivity continuum models on bulk DOC

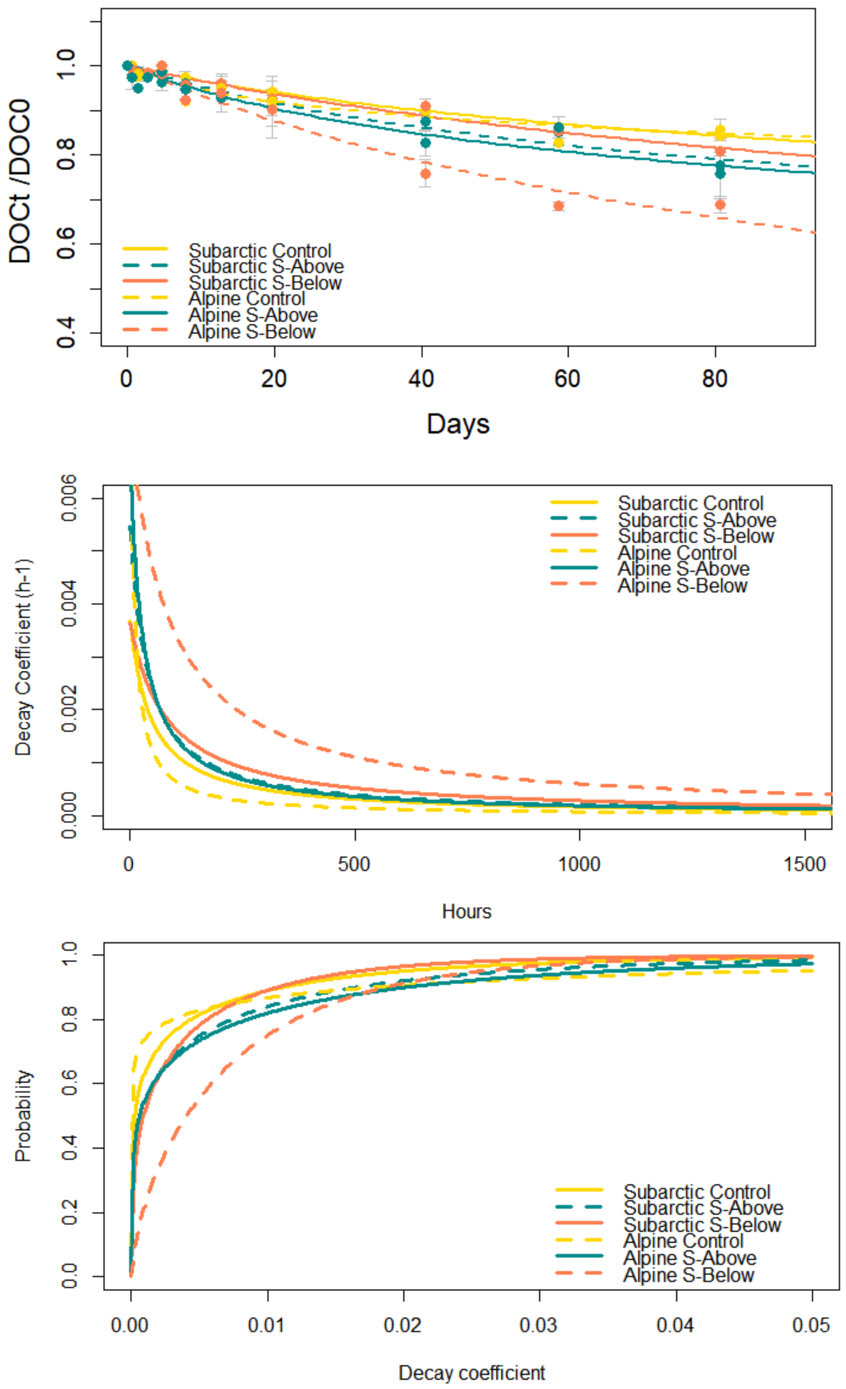

Supplementary Figure S3. Time series of the relative decrease in dissolved organic carbon (DOCt/DOC0) during the long-term incubations (a) Mean and standard error values are shown. The lines show the values predicted by the reactivity continuum model. Shown are modelled decay coefficients (d^{-1}) (b) and probability distribution of decay coefficients (b) obtained from the reactivity continuum modeling of bulk DOC concentration for the different treatments and at each location. Continuous and dashed lines correspond to the Subartic and Alpine lakes respectively. Blue represents the control, yellow the S-Above and grey the S-Below. Model parameters are provided in Supplementary Table S1.

Supplementary Table S1. Parameters of the reactivity continuum model of DOC degradation during long-term-incubations of samples amended with soil above (S-Above) and below (S-Below) the treeline and their corresponding, unamended lake water control in the subarctic and alpine lake.

	**Subarctic lake**			**Alpine lake**		
	Control	S-Above	S-Below	Control	S-Above	S-Below
α (days)[a]	45.88±1.61	38.32±1.72	83.02±1.73	8.84±2.67	28.43±3.11	88.95±2.33
ν[b]	0.169±0.009	0.209±0.016	0.301±0.039	0.715±0.002	0.189±0.025	0.65±0.28
k (d^{-1})[c]	0.0037±0.0002	0.0055±0.0005	0.0036±0.0005	0.0081±0.002	0.0067±0.0011	0.0073±0.0032

Values for the model parameters are predicted estimates and associated standard errors (SEs). SE for k correspond to the combined errors of α and ν

[a] average lifetime of the more reactive compounds

[b] relative preponderance of the most persistent compounds

[c] initial apparent decay coefficient

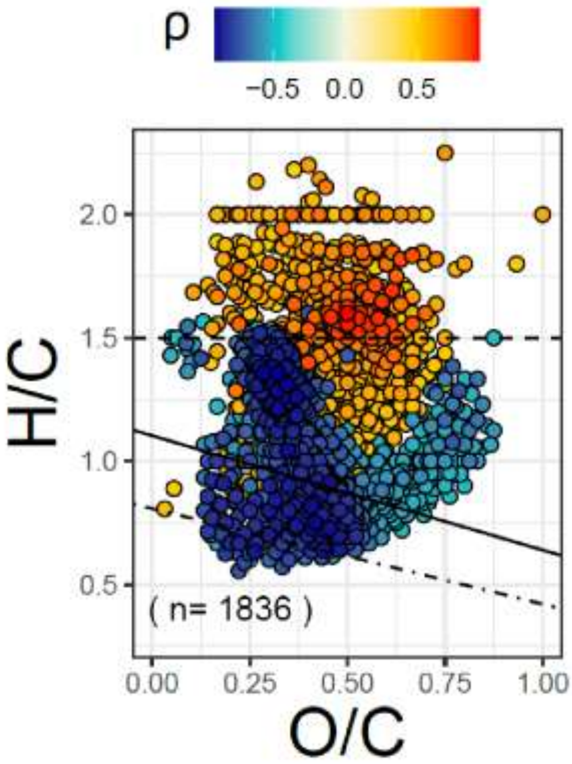

Supplementary Figure S4. Van Krevelen diagram depicting significant ($p<0.05$) Spearman rank correlations between individual compounds and bulk organic carbon decay rates. Rank correlations were corrected for multiple comparisons. Decay rates correspond to the output of the reactivity continuum models fit for the long-term incubations for the times sampled during the in-situ incubation (0, 6, 24 and 72 hours). Since individual compounds can overlap in Van Krevelen space, points are plotted in order by absolute values of ρ, such that stronger correlations are displayed on top.

Supplementary Information 3. FT-ICR MS data treatment

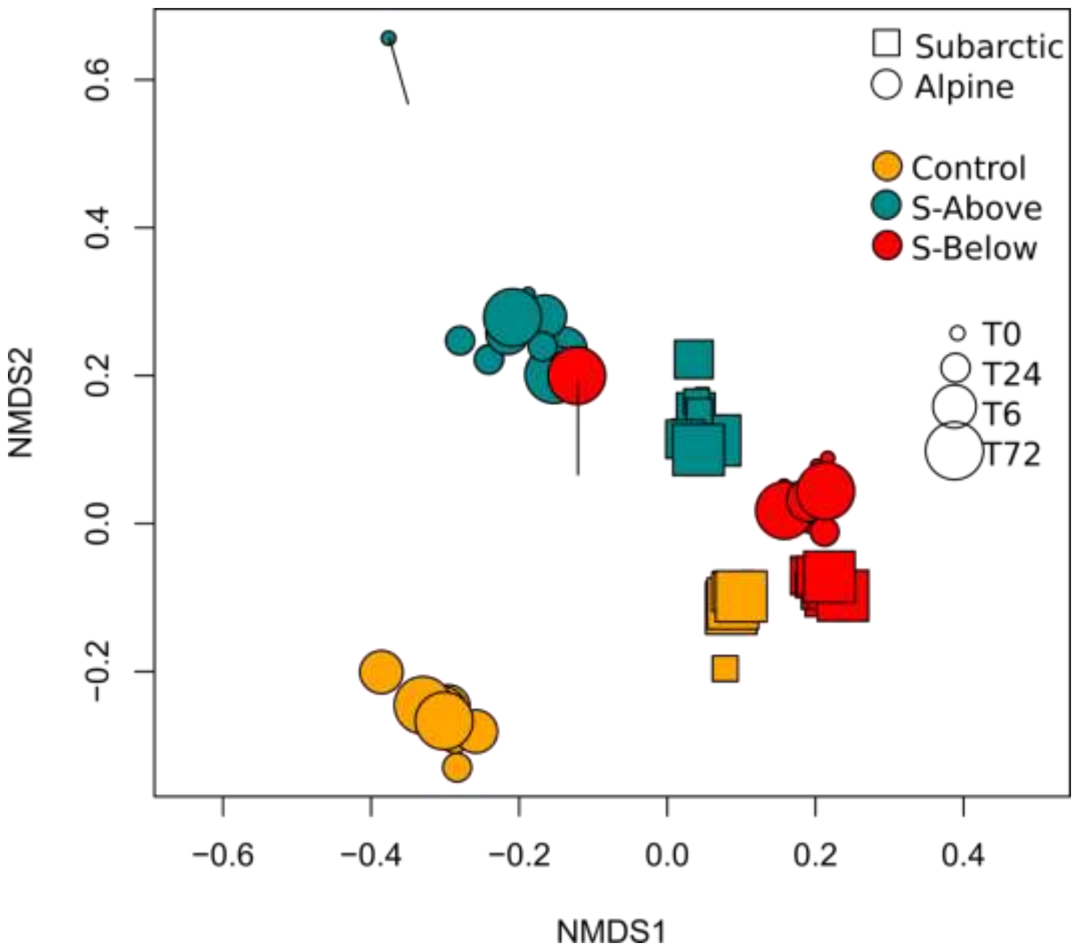

Supplementary Figure S5. Non-metric dimensional scaling (NMDS) on the Bray Curtis distances calculated for normalized peaks intensity. Stress= 0.084. For Subarctic samples, replicates A at times 0 and 72 h (indicated with an arrow) were considered outliers based on this result.

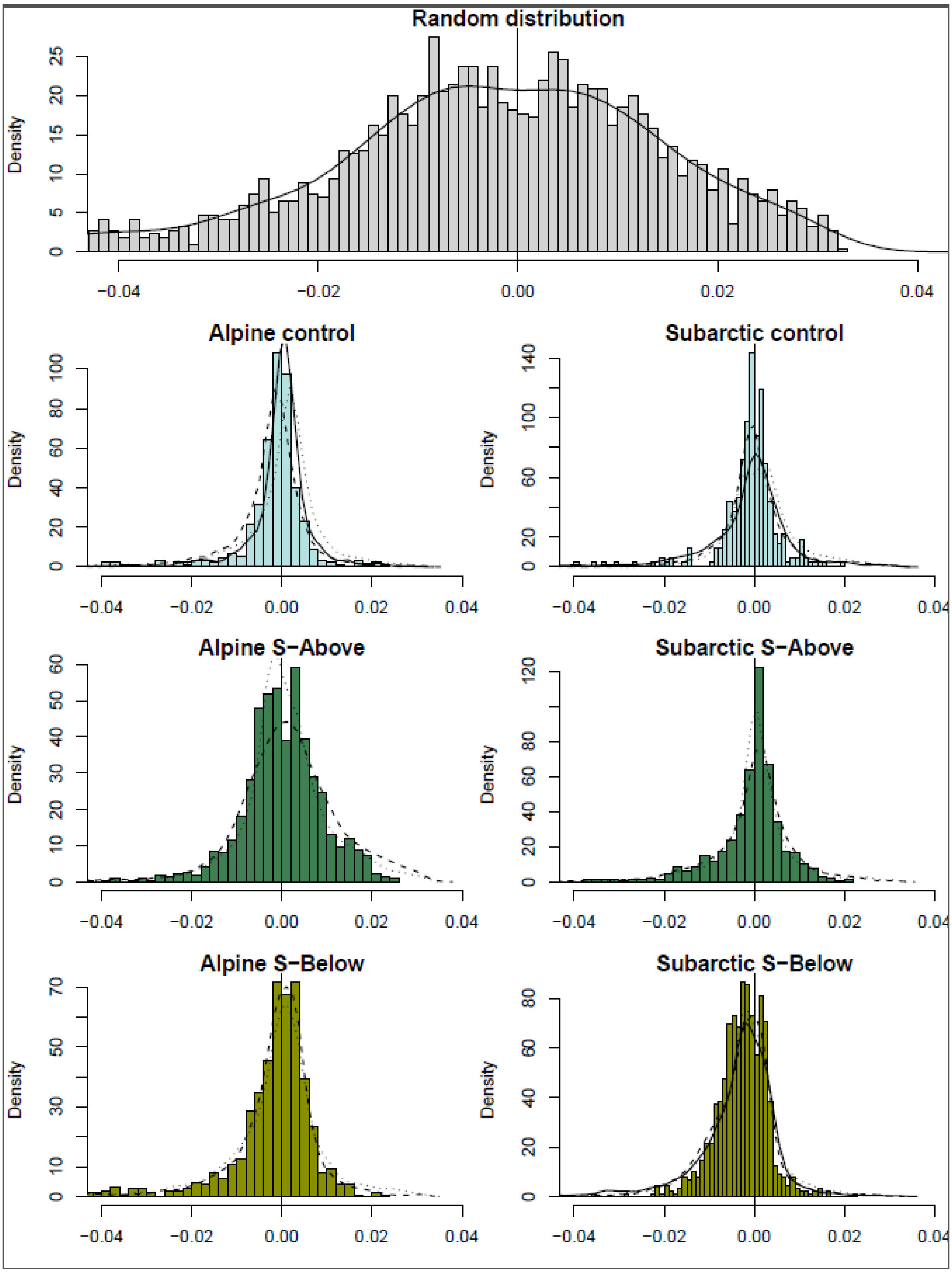

Supplementary Figure S6. Distribution of exponential coefficients k (h^{-1}) for the two lakes and three treatments. The distribution for the randomized dataset is also provided. The histogram corresponds to the first replicate available in all cases. Density lines for all replicates available for a given site and treatment are plotted, with replicate A being a solid line, replicate B a dashed line and replicate C a dotted line. Differences between distributions of each treatment and site were examined using non-parametric Kruskal–Wallis tests with a post-hoc multiple comparison Dunn's test (Supplementary Table S2).

Detail on methods: exponential fits on individual formulae

In order to assess the degradation and accumulation/production of each individual compound during the short-term incubation, we followed the procedure described by Kothawala and co-authors[11]. Briefly, according to the reactivity continuum model's underlying hypothesis[12], an exponential model was fitted to each individual compound using the gnls function of the nlme package in R. Model fit was evaluated according to its RMSE and the p-value of the fitted coefficient, models with RMSE > 0.358 and/or p-value > 0.05 were not considered. A more stringent criteria was used as the models were built upon four time points. The distribution of k obtained for each replicate was then tested against and idealized random distribution of intensities and only validated if those were significantly different using a Kolmogorov-Smirnoff test (ks.test; Supplementary Figure S3.2.).

Supplementary Table S2. Non-parametric Kruskal–Wallis tests with post-hoc comparisons (Dunn's test) on differences in distributions of k (Fig. S6) between sample pairs.

	**Alpine Control**	**Alpine S-Above**		**Subarctic Control**	**Subarctic S-Above**
**Alpine S-Above**	-3.14<br>p<0.01		**Subarctic S-Above**	-3.09<br>p<0.01	
**Alpine S-Below**	0.92<br>p=-0.18	3.70<br>p<0.01	**Subarctic S-Below**	2.04<br>p=0.02	4.91<br>p<0.01

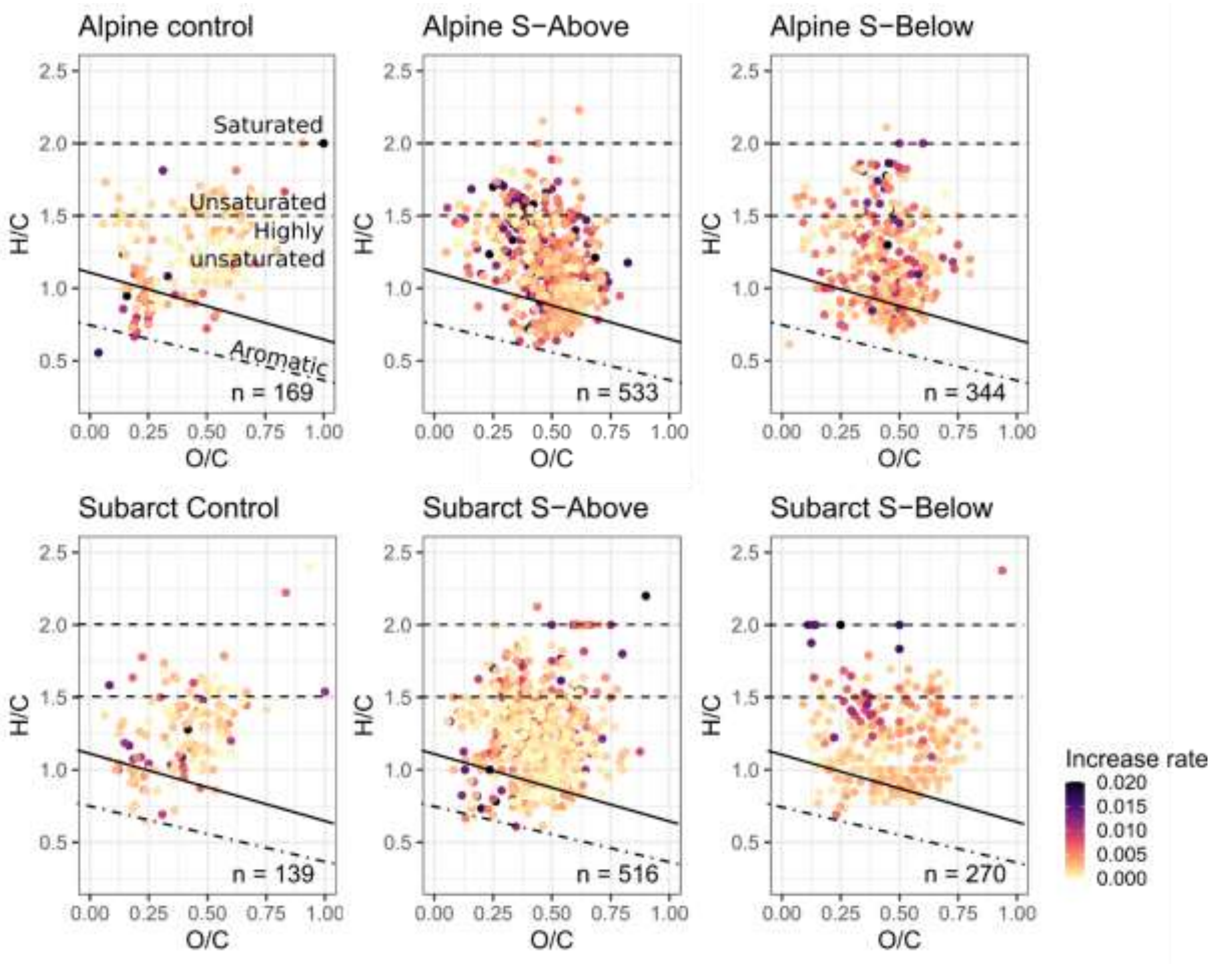

Supplementary Figure S7. Van Krevelen diagrams of compounds that increased over time in the different incubations. The color scale corresponds to the value of the decay coefficient for that compounds, with darker colors indicating compounds decaying faster.

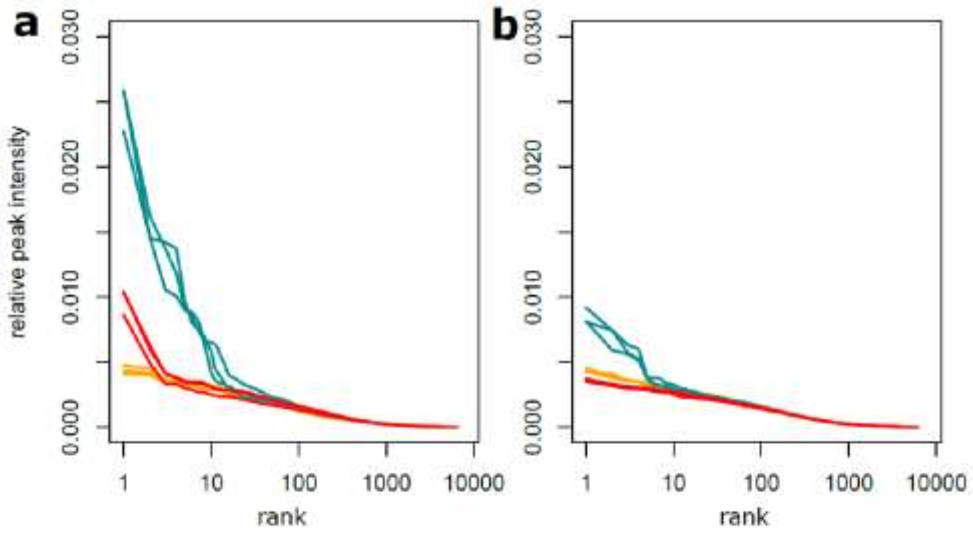

Supplementary Figure S8. Rank-intensity plots showing the distribution of relative intensity of control (orange), S-Above (turquoise) and S-Below (red) treatments at the initial timepoint in the Alpine (a) and Subarctic (b) lake. Note the log-scale of the y-axis and the few (~10) high-intensity compounds, particularly in the S-Above treatment.

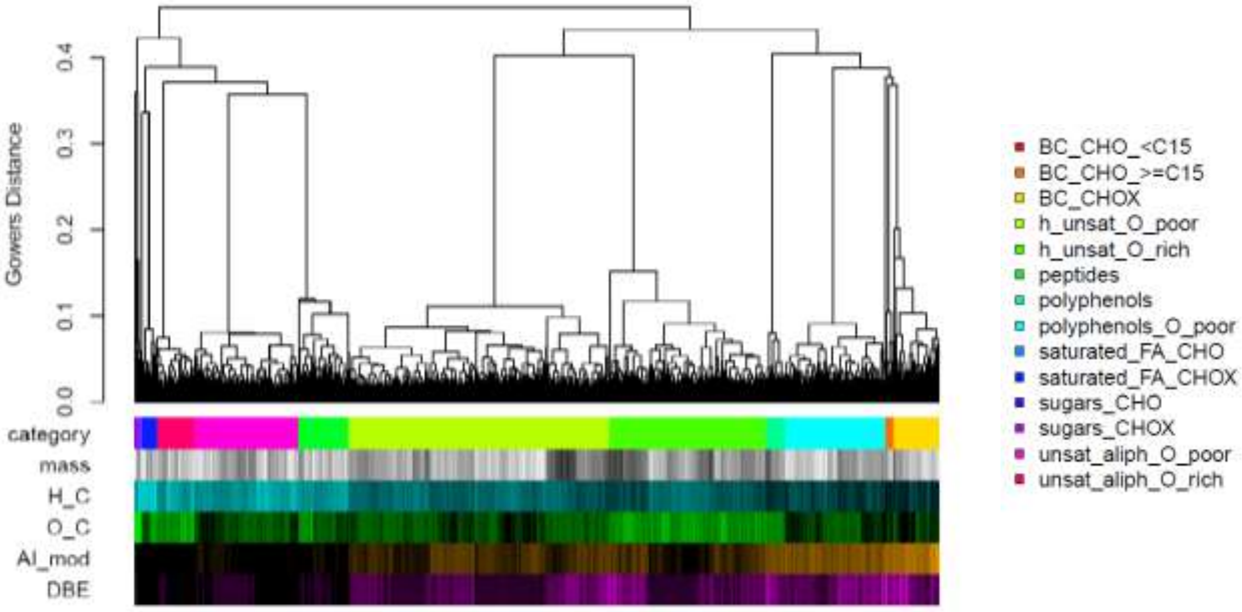

Supplementary Figure S9. Dendrogram of chemical similarity of all dissolved organic matter (DOM) compounds underlying chemodiversity estimation. Compound categories are color-coded (see legend) while continuous values (m/z, H:C, O:C, modified Aromaticity Index and Double Bond Equivalent) are show as colored gradients with low values being bright colors. For the calculation of chemodiversity, the total branch length in the dendrogram connecting 3000 randomly sampled DOM compounds was measured using the R function *treedive*.

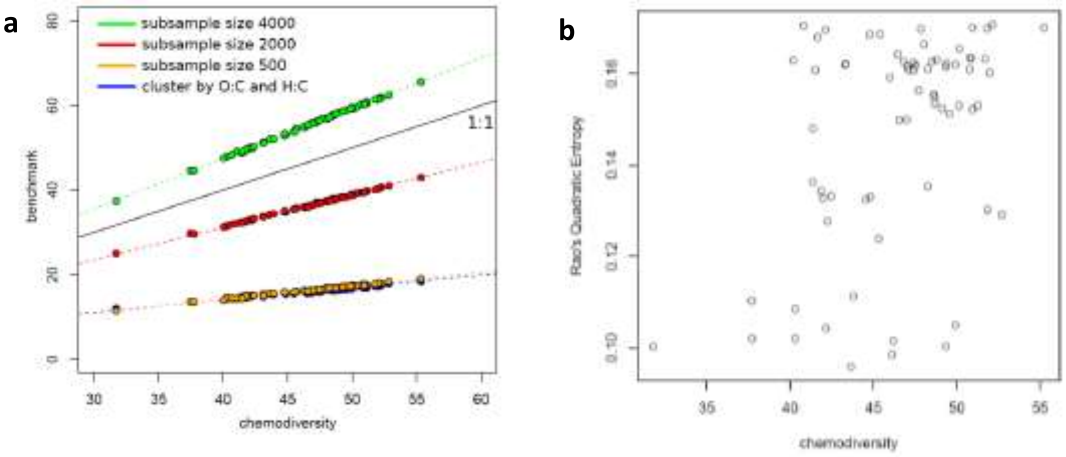

Supplementary Figure S10. Chemodiversity increased proportionately with increasing subsample size (a). The results for n=3000 (1:1 line) are reported in the main text. Panel b provides a comparison between the chemodiversity index and Rao's Quadratic Entropy measure of chemodiversity[13] which takes relative peak intensity into account.

Supplementary Information 4. Microbial respiration

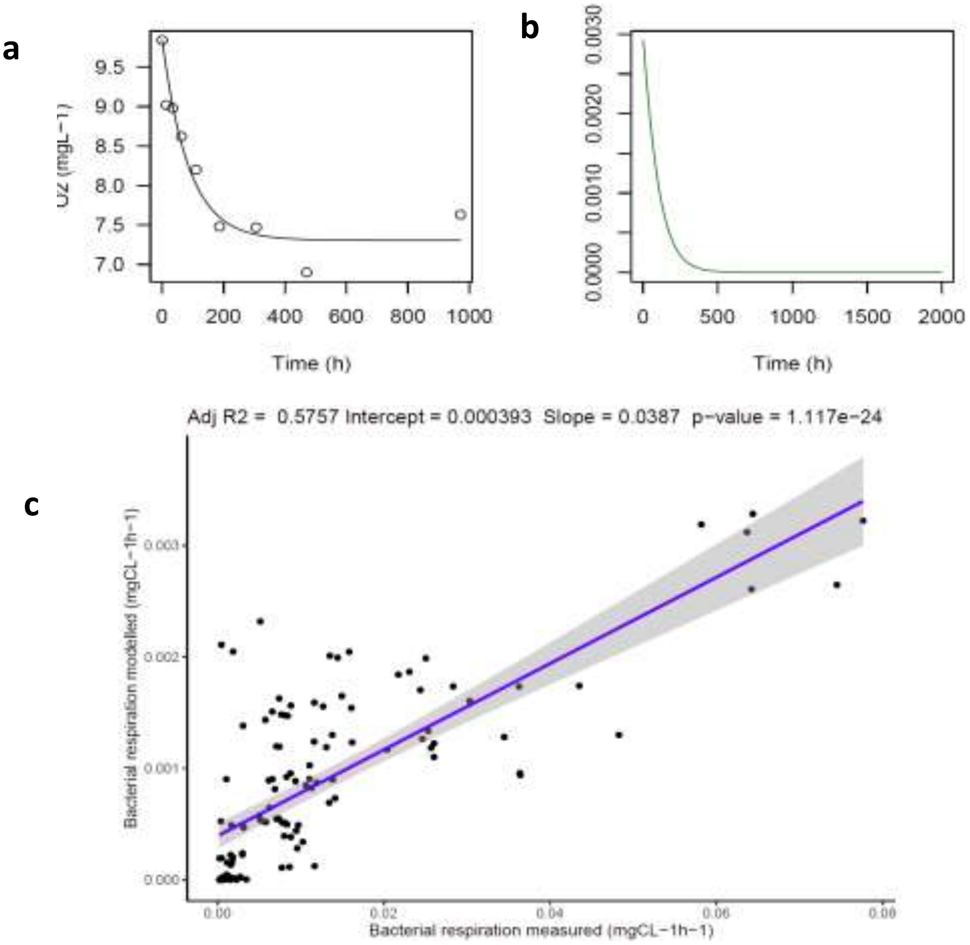

Supplementary Figure S11. Bacterial respiration rates calculation. Oxygen concentration was measured regularly during the incubation for the replicate 3 of the S-Above treatment of the Sub-Arctic site (a) using a Microx system (PreSens, Germany). The line corresponds to

the non-linear fit. Bacterial respiration rates were calculated in two different ways. *Method I:* Respiration rates were obtained for the same replicate as the slope at each point of the curve (b). Note that modelled values are shown to up to 2000 h. For this, we fitted a non-linear model to the oxygen concentration time series for each replicate using the *drm* function of the *drc* package in R. Then, we calculated the slope of each point of the model and extracted the values for the measured time points to obtain the corresponding instantaneous bacterial respiration rates. Additionally, for each oxygen measurement, the Microx system was maintained in the sample for up to 5 minutes (*Method II*). We fitted a linear model to the measured O_2 during these periods and considered the slope of these models as bacterial respiration rate of that time point. When comparing bacterial respiration rates obtained using the two methods, we found significant correlation between *Method I* and *Method II* (c). However, we also found a difference of one order of magnitude in terms of respiration rate magnitude between both methods. We believe this is an effect of the short measurement period used in *Method II*, which can lead to overestimation of oxygen consumption rates[14]. Moreover, when comparing the values with previous studies using similar setups[15,16,17], we find the rates obtained with *Method I* to fall within a similar range and thus use them preferentially in this study.

Detail on methods: Assessing differences between treatments in the long-term incubations.

Data from long-term incubations (bacterial respiration, production rates and optical spectroscopy parameters) was analyzed using linear mixed effects models (*lme* function from *nlme* R package). The model contained the linear and interaction terms of site (Sub-Arctic or Alpine) and treatment (Control, S_Above and S_Below) as well as time as a random effect. We included a first-order temporal autoregressive process and a variance function allowing for different variances of the response variable per level of fixed effect when needed. Models were checked using diagnostic residual plots and the significance of the fixed effects was tested using an anova against a null model containing only the random effect[18]. Collinearity was assessed by checking VIF values (*vif* function of the *car* R package). The final model structure was selected by model comparison.

Supplementary References

1. Lakowicz, J.R., 2006. Principles of Fluorescence Spectroscopy, 3rd ed.
2. Murphy, K.R., Stedmon, C.A., Graeber, D., Bro, R., 2013. Fluorescence spectroscopy and multi-way techniques. PARAFAC. Anal. Methods 5, 6557–6566. https://doi.org/10.1039/C3AY41160E
3. Murphy, K.R., Stedmon, C.A., Wenig, P., Bro, R., 2014. OpenFluor– an online spectral library of auto-fluorescence by organic compounds in the environment. Anal. Methods 6, 658–661. https://doi.org/10.1039/C3AY41935E
4. Murphy, K.R., Ruiz, G.M., Dunsmuir, W.T.M., Waite, T.D., 2006. Optimized parameters for fluorescence-based verification of ballast water exchange by ships. Environ Sci Technol 40, 2357-2362.
5. Yamashita, Y., Scinto, L.J., Maie, N., Jaffe, R., 2010. Dissolved Organic Matter Characteristics Across a Subtropical Wetland's Landscape: Application of Optical Properties in the Assessment of Environmental Dynamics. Ecosystems 13, 1006-1019.
6. Dainard et al., (2015) Photobleaching of fluorescent dissolved organic matter in Beaufort Sea and North Atlantic Subtropical Gyre, Mar. Chem., 177, 630-637

7. Kothawala, D. N., C. A. Stedmon, R. A. Muller, G. A. Weyhenmeyer, S. J. Kohler, and L. J. Tranvik. 2014. Controls of dissolved organic matter quality: Evidence from a large-scale boreal lake survey, Glob Chang Biol. 20, 1101–1114
8. Kowalczuk, P., M. J. Durako, H. Young, A. E. Kahn, W. J. Cooper, M. Gonsior, 2009. Characterization of dissolved organic matter fluorescence in the South Atlantic Bight with use of PARAFAC model: Interannual variability. Marine Chemistry 113, 182-196
9. Stedmon CA, Thomas DN, Papadimitriou S, Granskog MA & G Dieckmann (2011). Using fluorescence to characterize dissolved organic matter in Antarctic sea ice brines. J. Geophys. Res. 116(G03027) 1-9
10. Cuss, C. W., Guéguen C., 2013. Distinguishing dissolved organic matter at its origin: Size and optical properties of leaf-litter leachates, Chemosphere 92, 1483-1489
11. Mostovaya, A., Hawkes, J.A., Koehler, B., Dittmar, T., Tranvik, L.J., 2017. Emergence of the Reactivity Continuum of Organic Matter from Kinetics of a Multitude of Individual Molecular Constituents. Environmental Science & Technology 11571−11579. https://doi.org/10.1021/acs.est.7b02876
12. Koehler, B., Von Wachenfeldt, E., Kothawala, D.N., Tranvik, L.J., 2012. Reactivity continuum of dissolved organic carbon decomposition in lake water. Journal of Geophysical Research: Biogeosciences 117, 1–14.
13. Tanentzap, A.J., Fitch, A., Orland, C., Emilson, E.J.S., Yakimovich, K.M., Osterholz, H., Dittmar, T., 2019. Chemical and microbial diversity covary in fresh water to influence ecosystem functioning. PNAS 116, 24689–24695. https://doi.org/10.1073/pnas.1904896116
14. Pollard, P.C., 2013. In situ rapid measures of total respiration rate capture the super labile doc bacterial substrates of freshwater. Limnology and Oceanography: Methods 11, 584–593. https://doi.org/10.4319/lom.2013.11.584
15. Berggren, M., Lapierre, J.-F., del Giorgio, P. a., 2012. Magnitude and regulation of bacterioplankton respiratory quotient across freshwater environmental gradients. The ISME Journal 6, 984–993. https://doi.org/10.1038/ismej.2011.157
16. del Giorgio, P. a., Cole, J.J., 1998. Bacterial growth efficiency in natural aquatic systems. Annual Review of Ecology and Systematics 29, 503–541
17. Guillemette, F., del Giorgio, P. a., 2011. Reconstructing the various facets of dissolved organic carbon bioavailability in freshwater ecosystems. Limnology and Oceanography 56, 734–748. https://doi.org/10.4319/lo.2011.56.2.0734
18. Crawley, M.J., 2012. The R Book.
