# Supplementary material for: Treeline displacement may affect lake dissolved organic matter processing at high latitudes and altitudes
Source: Nat Commun. 2024 Mar 26;15:2640. doi: 10.1038/s41467-024-46789-5 (PMC10965997; doi:10.1038/s41467-024-46789-5)
Supplement: Supplementary file 3 — Reporting Summary [file 41467_2024_46789_MOESM3_ESM.pdf]

nature portfolio

Corresponding author(s): Hannes Peter

Last updated by author(s): Feb 26, 2024

Reporting Summary

Nature Portfolio wishes to improve the reproducibility of the work that we publish. This form provides structure for consistency and transparency in reporting. For further information on Nature Portfolio policies, see our Editorial Policies and the Editorial Policy Checklist.

Statistics

For all statistical analyses, confirm that the following items are present in the figure legend, table legend, main text, or Methods section.

n/a	Confirmed	
☐	☒	The exact sample size (*n*) for each experimental group/condition, given as a discrete number and unit of measurement
☐	☒	A statement on whether measurements were taken from distinct samples or whether the same sample was measured repeatedly
☐	☒	The statistical test(s) used AND whether they are one- or two-sided<br>*Only common tests should be described solely by name; describe more complex techniques in the Methods section.*
☐	☒	A description of all covariates tested
☐	☒	A description of any assumptions or corrections, such as tests of normality and adjustment for multiple comparisons
☐	☒	A full description of the statistical parameters including central tendency (e.g. means) or other basic estimates (e.g. regression coefficient) AND variation (e.g. standard deviation) or associated estimates of uncertainty (e.g. confidence intervals)
☐	☒	For null hypothesis testing, the test statistic (e.g. *F*, *t*, *r*) with confidence intervals, effect sizes, degrees of freedom and *P* value noted<br>*Give P values as exact values whenever suitable.*
☒	☐	For Bayesian analysis, information on the choice of priors and Markov chain Monte Carlo settings
☒	☐	For hierarchical and complex designs, identification of the appropriate level for tests and full reporting of outcomes
☒	☐	Estimates of effect sizes (e.g. Cohen's *d*, Pearson's *r*), indicating how they were calculated

Our web collection on statistics for biologists contains articles on many of the points above.

Software and code

Policy information about availability of computer code

Data collection	FT-ICR MS data was collected using Data Analysis Software v4.0 SP4 (Bruker Daltronics, Germany). Spectrophotometric data was collected using UV WinLab v7.1. Fluorometric data was collected using FluoroMax-4 (Horiba, Japan). DOC concentration was measured with Shimadzu TOC-L and Shimadzu TOL-VCPH.
Data analysis	Statistical analyses and Figures were prepared using R v 4.1.2. PARAFAC analyses and fluorometric data treatment were performed using Matlab and the FDOMcorr toolbox.

For manuscripts utilizing custom algorithms or software that are central to the research but not yet described in published literature, software must be made available to editors and reviewers. We strongly encourage code deposition in a community repository (e.g. GitHub). See the Nature Portfolio guidelines for submitting code & software for further information.

Data

Policy information about availability of data

All manuscripts must include a data availability statement. This statement should provide the following information, where applicable:

- Accession codes, unique identifiers, or web links for publicly available datasets
- A description of any restrictions on data availability
- For clinical datasets or third party data, please ensure that the statement adheres to our policy

We provide all raw data and R code for analysis at zenodo under doi: 10.5281/zenodo.10578476. The PARAFAC models were additionally uploaded to the OpenFluor database (https://openfluor.lablicate.com/) under accession 18555.

Research involving human participants, their data, or biological material

Policy information about studies with human participants or human data. See also policy information about sex, gender (identity/presentation), and sexual orientation and race, ethnicity and racism.

Reporting on sex and gender	NA
Reporting on race, ethnicity, or other socially relevant groupings	NA
Population characteristics	NA
Recruitment	NA
Ethics oversight	NA

Note that full information on the approval of the study protocol must also be provided in the manuscript.

Field-specific reporting

Please select the one below that is the best fit for your research. If you are not sure, read the appropriate sections before making your selection.

☐ Life sciences ☐ Behavioural & social sciences ☒ Ecological, evolutionary & environmental sciences

For a reference copy of the document with all sections, see nature.com/documents/nr-reporting-summary-flat.pdf

Ecological, evolutionary & environmental sciences study design

All studies must disclose on these points even when the disclosure is negative.

Study description	We experimentally manipulated organic matter in mesocosm incubations in-situ in two lakes to simulate the effects of the displacement of the treeline due to climate change. In both lakes, three treatment factors (soil-above, soil-below, control) with 3 independent replicates each were incubated and sampled periodically (4 time points). In total, 72 samples were processed. Long-term incubations were performed using the same sampling design (soil-above, soil-below and unamended controls in triplicates).
Research sample	The samples were water sampled from the incubations. We analysed the dissolved organic carbon composition and concentration in each of these water samples. These samples (i.e. populations) are meant to represent the lake water of the alpine and subarctic lake.
Sampling strategy	Samples and data were collected by the first and corresponding author. Samples were subsampled from the in-situ incubations into pre-cleaned glassware. No prior information regarding number of water samples was available, hence we opted for triplicate samples from our factorial (soil-above, soil-below, control) experiment. Samples were immediately acidified and concentrated using solid-phase extraction prior to FT-ICR MS analysis.
Data collection	FT-ICR Ms data was recorded by Data Analysis Software (Bruker). Spectrophotometric data was collected using the UV WinLab Software (Perkin Elmer). Spectrophotometric data was collected using UV WinLab v7.1. Fluorometric data was collected using FluoroMax-4 (Horiba, Japan). DOC concentration was measured with Shimadzu TOC-L and Shimadzu TOL-VCPH.
Timing and spatial scale	We performed the experiments in-situ in June 2014 (Lake Saanajaervi) and in August 2014 (Lake Gossenkoellesee). This is well within the ice-free season when the likelihood of transport of soil-derived organic matter is the largest (compared to other seasons when soils are frozen and precipitation falls mainly as snow). Temperature-dependent microbial activity is also expected to peak in this season. Samples were taken after 0.6, 24, and 72h, reflecting the immediate and short-term activity of microorganisms. Long-term incubations (81 days) were started at the same time and were sampled after 0, 0.5, 1, 3, 5, 7, 12, 40, 58 and 81 days. These incubations reflect the longer-term dynamics that can arise, for instance, from re-organization of microbial communities.

Data exclusions	two FT-ICR MS samples were identified as outliers (based on their molecular similarity and using multivariate statistical tools (shown in Supplementary Information) and removed.
Reproducibility	We used triplicate incubations to assess variation within treatments and across treatments (ANOVA design). No attempts to repeat the same experiments were performed.
Randomization	Randomization was not relevant in our study because samples were not grouped and all involved researchers participated similarly in experiments.
Blinding	Blinding was not relevant in our study because measurements were performed in an unbiased way using calibrated devices.

Did the study involve field work? ☒ Yes ☐ No

Field work, collection and transport

Field conditions	Experiments were performed during summer in Lake Saanajaervi and Lake Gossenkoellesee. Air temperatures were nevertheless low (between 5 and 15C) with pronounced diurnal variation due to the high latitude and altitude of the sites. Periods of stable good weather (i.e. no rain, heavy cloud cover) were chosen for both experiments.
Location	Lake Saanajaervi, 69°50N 20°870E, altitude 679 m a.s.l, size: 461 ha, max depth: 24m. Lake Gossenkoellesee: 47°130N 11°010E, altitude 2471 m a.s.l., area 1.7ha, max. depth: 9.9 m
Access & import/export	Both sites were reached by hiking. Experimental and sampling equipment had to be carried from the closest roads. Work at both sites was facilitated by research stations.
Disturbance	Given the microbial focus of the study, the in-situ experiments were rather small scale (i.e. floating platform of 1 x 1 m). The mesocosms were ankered to the lake bottom which represents a localized disturbance to the ecosystem. All experimental equipment was removed at the end of the experiments and no long-term disturbances are expected to arise from our work.

Reporting for specific materials, systems and methods

We require information from authors about some types of materials, experimental systems and methods used in many studies. Here, indicate whether each material, system or method listed is relevant to your study. If you are not sure if a list item applies to your research, read the appropriate section before selecting a response.

Materials & experimental systems

n/a	Involved in the study
☒	☐ Antibodies
☒	☐ Eukaryotic cell lines
☒	☐ Palaeontology and archaeology
☒	☐ Animals and other organisms
☒	☐ Clinical data
☒	☐ Dual use research of concern
☒	☐ Plants

Methods

n/a	Involved in the study
☒	☐ ChIP-seq
☒	☐ Flow cytometry
☒	☐ MRI-based neuroimaging

Plants

Seed stocks	NA
Novel plant genotypes	NA
Authentication	NA
